# Femoral Artery Thrombosis: Rare Sequelae of a Common Entity

**DOI:** 10.7759/cureus.32372

**Published:** 2022-12-10

**Authors:** Aditi Rawat, Bhavana B Lakhkar, Sagar Karotkar, Mahaveer S Lakra, Vaishnavi V Kantode, Mayur B Wanjari

**Affiliations:** 1 Department of Pediatrics, Jawaharlal Nehru Medical College, Datta Meghe Institute of Medical Sciences (Deemed to be University), Wardha, IND; 2 Department of Pediatrics and Neonatology, Jawaharlal Nehru Medical College, Datta Meghe Institute of Medical Sciences (Deemed to be University), Wardha, IND; 3 Department of Medical Surgical Nursing, Srimati Radhikabai Meghe Memorial College of Nursing, Datta Meghe Institute of Medical Sciences (Deemed to be University), Wardha, IND; 4 Department of Research, Jawaharlal Nehru Medical College, Datta Meghe Institute of Medical Sciences (Deemed to be University), Wardha, IND

**Keywords:** acute kidney injury, thrombosis, gangrene, hypernatremic dehydration, neonate

## Abstract

Neonatal hypernatremic dehydration (NHD) is a common complication in breastfed neonates which if not recognized early can lead to life-threatening complications. Only a few cases of NHD leading to peripheral gangrene have been reported in the literature. We report a case of a 14-day-old neonate with complaints of dyspnoea, poor oral intake, and gangrenous changes in the left leg. There was a 28% weight loss since birth due to inadequate feeding. The baby was diagnosed with severe hypernatremic dehydration with Kidney Disease Improving Global Outcomes (KDIGO) stage 4 acute kidney injury requiring peritoneal dialysis. Ultrasonography of the left lower extremity revealed a distal femoral artery thrombus leading to dry gangrene requiring amputation. There were neurological signs like altered sensorium and drug-resistant seizures which were suspicious for intracranial pathology like cerebral venous sinus thrombosis. Prevention and early diagnosis of NHD are essential to prevent the occurrences of such grave complications. It can be easily achieved by improving the vigilance regarding the adequacy of feeds subjectively by the mother if the baby is at home and objectively by physicians in the hospital setting. These simple interventions have the potential to prevent readmissions due to not only simple feeding complications but grave complications as mentioned above as well and save precious lives.

## Introduction

Exclusively breastfed newborns have relatively high blood sodium levels than those who receive formula feed [1]. In solely breastfed newborns, hypernatremic dehydration is linked to a free water deficit brought on by insufficient fluid intake. In primary care practice, it is a prevalent but underappreciated issue because in vivo fluid movement can cause the degree of dehydration to be underestimated [2]. The incidence of hypernatremia can range from 1 to 5.6% in term neonates and up to 40% in preterm neonates [3].

Neonatal hypernatremia can lead to many detrimental complications like intracranial hemorrhage, cerebral edema, acute kidney injury, transaminitis, and disseminated intravascular coagulation. It could also infrequently cause venous and arterial thrombosis, resulting in cerebral edema and intracranial hemorrhage [4]. Rare reports of neonatal hypernatremic dehydration (NHD) with thrombosis have been documented involving the abdominal aorta, cerebral venous sinus, retinal artery, and renal vein [5]. The incidence of peripheral thrombosis is only 1% in NHD cases [6]. Here we are reporting femoral artery thrombosis as a rare complication of NHD.

## Case presentation

**Figure 1:**
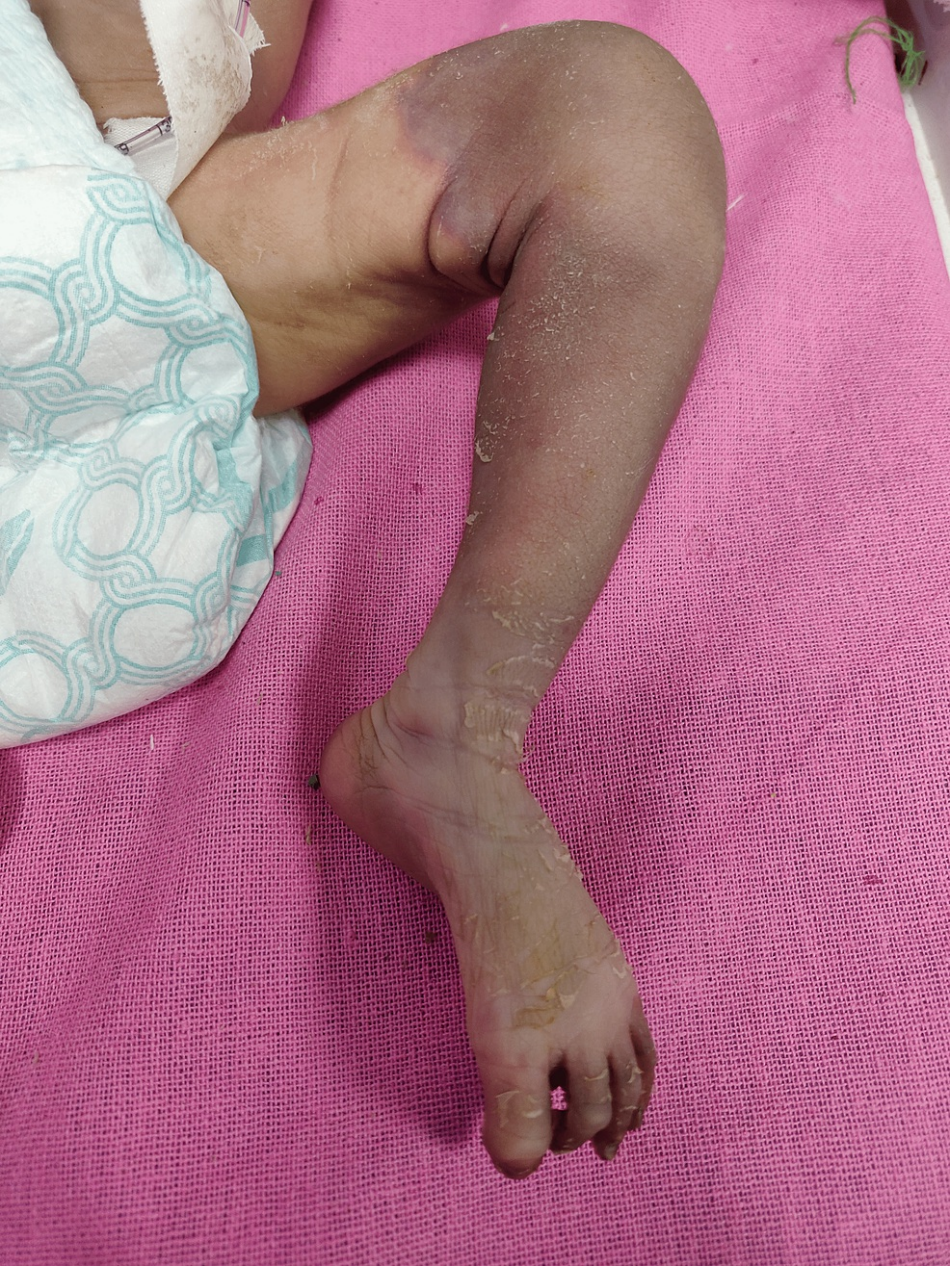
Shows dry gangrene of the left leg with a demarcation line.

A 3.5 kg male child was born to a multigravida mother at 37 weeks of gestational age via normal vaginal delivery. The immediate postnatal period was uneventful, and the baby was discharged on the third day of life. The baby had poor oral intake and was breastfed only 4-6 times in 24 hours. On the eighth day of life, the baby had febrile episodes for three days following which he had an episode of generalized tonic-clonic seizure for which anticonvulsants were started in the nearby hospital. The patient came to our hospital on the 14th day of life with complaints of dyspnoea, poor oral intake, and gangrenous changes in the left leg. On examination baby was lethargic, heart rate = 170 beats per minute (tachycardia), respiratory rate was 65 breaths per minute (tachypneic), temp = 35 °C (hypothermic), and weight = 2500 g (28% loss of birth weight). The oxygen saturation was below 90% on room air. There were frank signs of dehydration like depressed anterior fontanel, sunken eyes, dry lips, and doughy texture of the skin. Dry gangrene was noted on the left lower limb with no palpable pulses in the tibial, popliteal arteries, and dorsalis pedis artery (Figure 1).

The patient was put on continuous positive airway pressure (CPAP) for respiratory distress, and intravenous antibiotics were started. Initial blood reports showed sodium 175 meq/L, potassium 5.8 meq/L, urea 477 mg/dl, and creatinine 7.9 mg/dl were suggestive of severe hypernatremic dehydration with pre-renal acute kidney injury. After saline bolus, a slow intravenous correction was started for hypernatremia. The baby was euglycemic with no hemoconcentration as the hematocrit was 56.3%. The septic screen was negative, and the blood culture had no growth. Thrombocytopenia with a platelet count of 25,000 cells/dl was present, for which a platelet transfusion was given. The coagulation profile was normal with an international normalized ratio (INR-1.28) and the D-dimer was 529 ng/ml. Doppler ultrasound of the left lower limb was suggestive of distal femoral artery thrombus for which subcutaneous enoxaparin was started. Protein C, protein S, and factor V Leiden mutation testing was negative. The baby was mechanically ventilated for deteriorating sensorium and multiple seizures despite multiple antiepileptics. Cranial ultrasound was inconclusive.

The baby developed anuria over the next 12 hours with Kidney Disease Improving Global Outcomes (KDIGO) stage 4 acute kidney injury for which he underwent peritoneal dialysis. Renal function parameters started improving thereafter. The renal parameters at the end of dialysis were urea-120 mg/dl, creat-5.5mg/dl, sodium-142 meq/L, and potassium-3.4meq/L.

The limb gangrene continued to worsen, for which a plastic surgeon recommended amputation. The baby became comatose and despite all the management, went into cardiac arrest on the seventh day of admission. This baby is hypothesized to have had cerebral venous sinus thrombosis as well, which led to seizures, altered sensorium, and eventually death.

This case depicted a rare complication of hypernatremia dehydration with an arterial thrombus of the lower limb with dry gangrene requiring amputation. Cerebral venous sinus thrombosis, a known complication of NHD, needs confirmation and magnetic resonance imaging (MRI) angiography, which could not be done due to the moribund status of the baby. In conclusion, hypernatremia dehydration can have life-threatening complications, and adequate postnatal feeding has an immense role in preventing it in the first place.

## Discussion

Hypernatremia is defined as serum sodium levels > 145 mEq/L. Serum sodium levels between 145 and 149 mEq/L are considered mild hypernatremia; 150-160 mEq/L is moderate hypernatremia, and above 160 mEq/L is severe hypernatremia with a 66% mortality in severe NHD [7]. Vomiting, diarrhea, incorrect preparation of infant formula, infrequent breastfeeding, and lactation failure are significant contributors to this illness in neonates. It usually presents between the first and third week of life. Clinical presentation is diverse in the form of weight loss, hyperbilirubinemia, irritability, lethargy, seizures, decreased urine output, and poor feeding [8].

Most problems, particularly seizures, arise during sodium correction. Rehydrating the infant gradually is understood to be the cornerstone of therapy. Rapid attempts to reduce the excessive sodium concentration carry a significant danger of osmotic alterations in the newborn infant's brain, which could increase cerebral edema and result in severe brain damage [9]. Neonatal limb gangrene is itself an uncommon entity usually predisposed by prematurity, sepsis, inherited hypercoagulable state, umbilical artery cannulation, arterial thrombosis, polycythemia, hyperglycemia, perinatal asphyxia, and maternal diabetes [10]. Hypernatremia can lead to hyperviscosity which can make the patient prone to venous or arterial thrombosis.

Our patient had severe hypernatremia due to inadequate breastfeeding as shown by drastic weight loss leading to hyperviscosity causing femoral artery thrombosis with dry gangrene. There are very few reports to date highlighting limb gangrene as a consequence of hypernatremia. Morin and Chevalier reported a case of an exclusively breastfed neonate with severe hypernatremic dehydration presenting with disseminated intravascular coagulation and right lower limb gangrene requiring amputation of all five toes and surgical debridement of the metatarsals secondary to inadequate feeds [11].

Tamene et al. reported a case of a 12-day-old neonate with the nose, upper lip, soft and hard palate, symmetric lower limb, and fifth finger gangrene due to severe hypernatremic dehydration complicated by disseminated intravascular coagulation [12]. Similarly, Shroff et al. demonstrated a case series of five neonates with NHD out of which three babies had vascular thrombus of major vessels at the time of presentation [13].

All of the cases had a common etiology of exclusive and inadequate breastfeeding presenting with significant weight loss similar to our case. This emphasizes the importance of parent and physician vigilance in ensuring the adequacy of breastfeeding. In our case, the non-continuation of proper feeding after the third day of life led to such catastrophic events. Since hypernatremic dehydration can cause lifelong disability or can be fatal in severe cases, its prevention is crucial. It is necessary to schedule regular follow-up visits with the mother and the newborn child to reinforce effective breastfeeding and to identify any infant problems at an earlier stage.

## Conclusions

Professionals need to be aware that an exclusively breastfed infant may develop hypernatremic dehydration. Even without routine testing for sodium levels and renal parameters, simple monitoring for weight loss, parent education on red flag signs of dehydration, and signs of suboptimal feeding can prevent a majority of such cases. Early detection and prevention are required to avoid the development of complications. Lactation assistance should be provided to ensure successful breastfeeding. This case showed a rare complication of hypernatremia dehydration with an arterial thrombus of the lower limb with dry gangrene requiring amputation.
